# Foliar Supplied Boron Can Be Transported to Roots as a Boron-Sucrose Complex via Phloem in Citrus Trees

**DOI:** 10.3389/fpls.2020.00250

**Published:** 2020-03-10

**Authors:** Wei Du, Zhi-Yong Pan, Syed Bilal Hussain, Zhong-Xing Han, Shu-Ang Peng, Yong-Zhong Liu

**Affiliations:** ^1^Key Laboratory of Horticultural Plant Biology, Ministry of Education, Huazhong Agricultural University, Wuhan, China; ^2^College of Horticulture & Forestry Sciences, Huazhong Agricultural University, Wuhan, China

**Keywords:** citrus, foliar boron, girdling, shading, phloem transport, sucrose

## Abstract

Although foliar boron (B) fertilization is regarded as an efficient way to remedy B deficiency, the mechanisms of foliar B transport from leaves to roots are still unclear. In this study, performed with 1-year-old “Newhall” navel orange (*Citrus sinensis*) grafted on trifoliate orange (*Poncirus trifoliata*) plants, we analyzed the B concentration in leaves and roots, B-sucrose complex in the phloem sap after foliar application of ^10^B, girdling, and/or shading treatments. Results indicated that ^10^B concentration was significantly increased in roots after foliar ^10^B treatment. On the other hand, both girdling the scion stem and shading over the plants with a black plastic net significantly reduced the B and ^10^B concentration in roots. LC-MS analysis revealed that foliar ^10^B-treated plants had higher concentration of sucrose and some sugar alcohols in the phloem sap as compared to foliar water-treated plants. Combining with the analysis in the artificial mixture of B and sucrose, a higher peak intensity of the ^10^B-sucrose complex was found in the phloem sap of foliar ^10^B-treated plants compared to the control plants. Taken together, it is concluded that foliar B can be long distance transported from leaves to roots via phloem, at least by forming a B-sucrose complex in citrus plants.

## Introduction

Boron (B) is a micronutrient that plays a pivotal role in cell wall stability (Chormova et al., 2014), photosynthesis (Wang et al., 2015), and carbon metabolism (Mishra and Heckathorn, 2016) in the plant. Thus, B deficiency inhibits plant growth, hinders leaf expansion, causes leaf chlorosis or shoot tip dieback, deforms leaf, flower, or fruit, decreases yield and fruit quality, limits root elongation (Wang et al., 2015). Preventing boron deficiency is critical to maintain crop yield and quality, and its success depends on the understanding of B transport and distribution mechanisms.

In general, plants absorb B mainly by roots from the soil in the form of boric acid. Then, B moves through the xylem to be distributed in different tissues or organs for utilization (Marschner et al., 1996; Takano et al., 2008). The occurrence of B deficiency is mainly caused by the low level of soluble B in the soil and/or low B utilization by plants (Wang et al., 2015). Soil B application is a common practice in commercial agriculture to prevent B deficiency (Shireen et al., 2018). Moreover, extensive efforts were devoted in the last several decades for assessing the mechanisms of B absorption, acropetal transport, and distribution (Takano et al., 2008; Miwa and Fujiwara, 2010; Reid, 2014; Yoshinari and Takano, 2017), expecting to improve the plant B-utilizing capability.

On the other hand, foliar B fertilization can be an efficient way to overcome B deficiency and ensure fruit yield and quality (Fregoni et al., 1978; DeMoranville and Deubert, 1987; Boaretto et al., 2011; Al-Obeed et al., 2018; Shireen et al., 2018). Previous studies showed that foliar B application can improve B availability in leaves and increase the B concentration in buds (Eichert and Goldbach, 2010; Liu et al., 2012). Moreover, foliar B can be transported from lower leaves to upper leaves or from mature leaves to reproductive organs via the phloem (Huang et al., 2001; Stangoulis et al., 2001; Huang et al., 2008; Liu et al., 2012). However, the efficiency of foliar B application to overcome B deficiency depends on B mobility through the phloem (Brown and Shelp, 1997), which appears to be linked to the possibility that B is complexed with certain metabolites (Reid, 2014). These metabolites may correspond to polyols (Lehto et al., 2004; Will et al., 2011) and/or sucrose (Stangoulis et al., 2001, 2010). The mobility of B in the phloem is high in many fruit species such as olive, apple, and peach through the formation of B-polyol complexes (Bieleski, 1982; Brown and Shelp, 1997). In fact, B is highly mobile in most species belonging to Oleaceae and Rosaceae families, which translocate large amounts of sugar alcohols in the phloem (Hu et al., 1997), while B shows low mobility in wheat and canola that translocate sucrose in the phloem (Stangoulis et al., 2010). Citrus is a vascular plant with sucrose being the main photosynthate transported in the phloem (Dinant et al., 2010; Hijaz and Killiny, 2014; Liu et al., 2015). However, the role of sucrose in B mobility from leaves to roots is unclear in citrus species.

*Citrus* is one of the world’s major fruit crops (Liu et al., 2012), which grows in more than 140 countries with the global production of over 146 million metric tons in 2017 (FAO, 2019). However, citrus is very sensitive to B deficiency and its major growing regions contain low levels of soluble B (Guidong et al., 2011; Wang et al., 2014, 2016). Many studies focused on citrus response to B deficiency or toxicity (Sheng et al., 2009; Boaretto et al., 2011; Mei et al., 2011; Liu et al., 2013; Zhou et al., 2014; Dong et al., 2016; Wu et al., 2018), and the molecular mechanisms for rootstock B-utilizing efficiency (An et al., 2012; Cañon et al., 2013; Zhou et al., 2015; Martínez-Cuenca et al., 2019). In the citrus industry, on the other hand, foliar application of B is an alternative way to supply B because foliar B sprays can be easily applied and may be rapidly absorbed by the foliage. However, whether the foliar-applied B can be transported to the root and what is the transporting form are still unclear in citrus species. Hence, in this study, ^10^B was used to trace the translocation of foliar applied B under both B-sufficient and -deficient conditions, while girdling was used to trace B transport mechanisms. Because shading with a black net can decrease leaf photosynthetic capacity (Correia et al., 2006), shading was then used to detect the possible role of sucrose in the B long-distance transport. Moreover, component analysis of the phloem sap was conducted to confirm this possibility. It is concluded that foliar supplied B can be long distance transported to citrus roots through the phloem as a B-sucrose complex.

## Materials and Methods

### Plant Materials and Treatments

**FIGURE 1:**
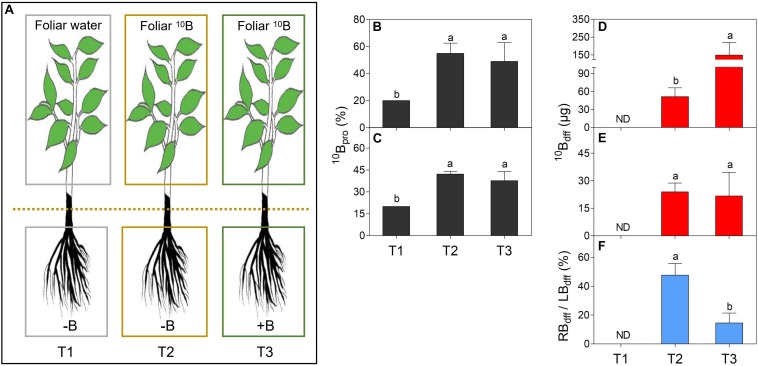
Comparison of proportion of ^10^B (^10^B_pro_) and ^10^B concentration derived from the foliar ^10^B (^10^B_df__f_) in leaves **(B,D)** and roots **(C,E)**, and the ratio of root ^10^B_dff_/leaf ^10^B_dff_ (RB_dff_/LB_dff_),**(F)** of different treatments **(A)**. Figure **(A)** shows three treatments: gray boxes refer to foliar application of ultra-pure water to B deficient seedlings as treatment 1 (T1); yellow boxes refer to foliar ^10^B application to B deficient seedlings as treatment 2 (T2); green boxes refer to foliar ^10^B application to B sufficient seedlings as treatment 3 (T3). Different lower-case letters indicate the significant difference between treatments at *P* < 0.05 as determined by the Duncan’s multiple range test.

**FIGURE 2:**
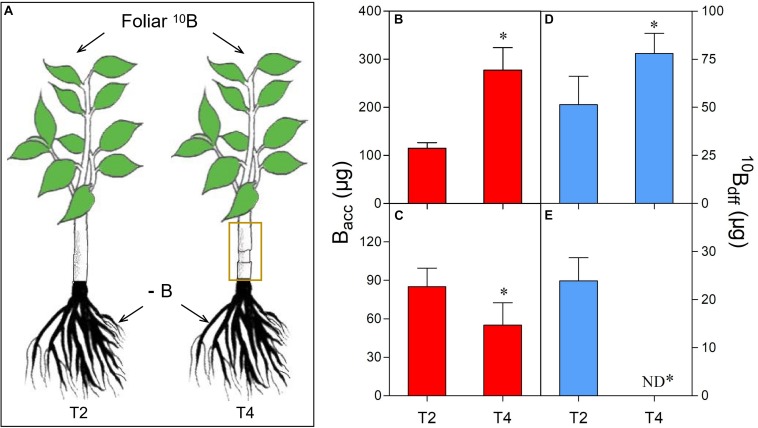
Comparison of B accumulation (B_acc_) and ^10^B concentration derived from foliar ^10^B (^10^B_df__f_) in leaves **(B,D)** and roots **(C,E)** of different treatments **(A)**. The yellow box refers to girdling the stem of foliar-^10^B treated plants as treatment 4 (T4). “^∗^” in each graph indicates significant differences between T2 and T4 treatments (*t*-test, *n* = 4, *P* < 0.05).

**FIGURE 3:**
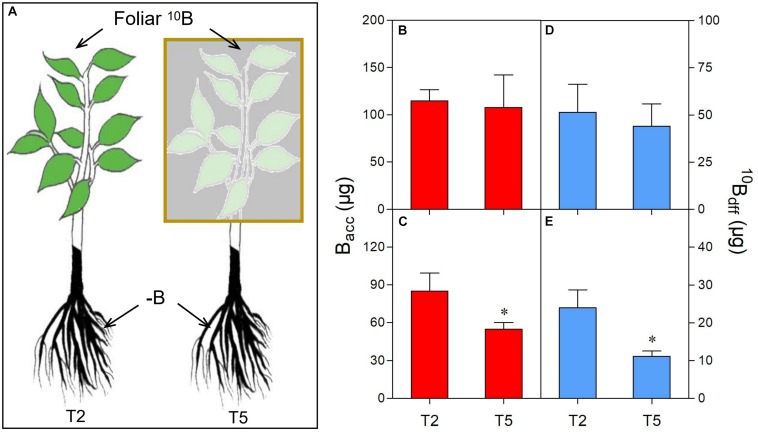
Comparison of B accumulation (B_acc_) and ^10^B concentration derived from foliar ^10^B (^10^B_df__f_) in leaves **(B,D)** and roots **(C,E)** of different treatments **(A)**. The yellow box refers to shading the foliar-^10^B treated plants as treatment 5 (T5). “^∗^” in each graph indicates significant differences between T2 and T5 treatments (*t*-test, *n* = 4, *P* < 0.05).

One-year-old “Newhall” navel orange (*Citrus sinensis* cv. Newhall) plants (*n* = 40), grafted on trifoliate orange (*Poncirus trifoliata*) rootstock, were individually transplanted to five liter-lightproof pots (one-pot contained one plant) with B-free quartz sand and perlite (1:1, v/v). These plants were placed in a greenhouse under natural sunlight conditions at Huazhong Agricultural University, Wuhan, China (Supplementary Figure S1). They were supplied with 3 liters of modified Hoagland No. 2 nutrient solution without B (Hoagland and Arnon, 1950) for 2 months at an interval of 1 week. Then, 20 plants with similar stem diameter, plant height and vigor were selected for five treatments (T1–T5): T1 (considered as control)- the upper and lower sides of all leaves were evenly wiped twice by cotton swabs which were saturated with ultra-pure water (plus 0.01% Tween-20) (Figure 1A), T2- the upper and lower sides of all leaves were evenly wiped twice by cotton swabs which were saturated with ^10^B (47 mM H_3_^10^BO_3_, 99% atom ^10^B, Aldrich, United States) solution (plus 0.01% Tween-20) (Figure 1A), T3- the plant leaves were treated like T2 plant leaves but the plants were irrigated with sufficient B (0.25 mg/L H_3_BO_3_) in sand culture (Figure 1A), T4- the plant leaves were treated like T2 plant leaves but the plant stems were girdled (8–10 mm wide at about 2 cm above the graft union) (Figure 2A), T5- the plant leaves were treated like T2 plant leaves but the plants were shaded with a black plastic net (the transmittance is 10.55%) (Figure 3A). All treatments were conducted in the same greenhouse (temperature≈30°C; relative humidity≈80%) at 9:00 a.m. Each treatment was replicated four times (one plant as one replication). Before treatment, new twigs were removed and 15–18 healthy leaves were retained per plant.

Seven weeks after treatment, roots and leaves of each plant in T1–T5 treatments were collected. After careful cleaning, they were dried in an oven and then used for B and ^10^B analysis. In addition, plant scion stems from T1- and T2-treatment were collected for phloem sap extraction.

### B Concentration and ^10^B Analysis

About 0.30 g dry samples were ashed in a muffle furnace at 500°C for 6 h. Afterward, they were dissolved in 30 mL of 1% HNO_3_. Half of the solution was used to measure B concentration (B_con_, μg/g) by an inductively coupled plasma-optical emission spectrometer (ICP-OES) (Bryson et al., 2014). The B accumulation (B_acc_) of given plant tissue was calculated according to the following formula.

B(μg)acc=B(μg/g)con×dryweight(g)

The remaining solution was used to determine the proportion of ^10^B (^10^B_pro_,%), by an inductively coupled plasma-mass spectrometer (ICP-MS). Because any tissue in the plant contains a background ^10^B, the ^10^B concentration derived from foliar ^10^B (^10^B_dff_, μg) was calculated according to the following formula (Liu et al., 2012).

B10(μg)dff=(B10-proc)1/(c-2c)1×B(μg/g)con×

d⁢r⁢y⁢w⁢e⁢i⁢g⁢h⁢t⁢(g).

c_1_ refers to the background ^10^B_pro_ which was determined from the control samples and the value was 20 ± 0.02% in the current study, and c_2_ refers to the ^10^B_pro_ in H_3_^10^BO_3_ in which the value was 99%.

### Phloem Sap Extraction

Phloem sap was collected as described by Hijaz and Killiny (2014). Briefly, scion stems were collected from T1- and T2-treatment plants and washed three times with ultra-pure water to remove dirt and residues. Then, clean stems of each plant were cut into 3–5 cm length pieces with sterile scissors. The bark was peeled off by sterile tweezers and then rinsed with ultra-pure water to clean xylem residues. Subsequently, the clean epidermis was cut into small pieces, mixed and transferred into a 0.5 mL centrifuge tube with punctured at the bottom. Each tube was packed tightly and then put into a 2 mL centrifuge tube for centrifugation. After centrifugation, about 40 μL of phloem sap was collected.

### Metabolite Extraction and Sample Preparation

About 30 μL phloem sap from each plant were thoroughly mixed with 800 μL cold mixture of methanol and acetonitrile (v/v, 1:1). Then, the mixture was processed with sonication for 1 h in an ice bath and then incubated at −20°C for 1 h. After centrifugation (14000 *g*, 20 min, 4°C), the supernatants were collected and then dried under vacuum. The lyophilized powder was re-dissolved with 50% acetonitrile, and then vortexed for 1 min. After centrifugation (14000 *g*, 15 min, 4°C), the supernatants were collected for LC-MS analyses.

Additionally, to ensure the data quality for metabolic profiling, quality control (QC) samples were prepared by pooling aliquots of all representative phloem saps. QC samples in each batch were prepared and analyzed as the experiment samples.

### Non-targeted Metabolites Analysis

Non-targeted metabolites, multivariate statistical and metabolites identification were performed according to the previous method (Wang et al., 2017). Metabolomics profiling was analyzed by using a UPLC-ESIQ-TOF-MS system (UHPLC, 1290 Infinity LC, Agilent Technologies, Santa Clara, CA, United States) coupled with Triple TOF 5600 (AB Sciex, Framingham, MA, United States). The detailed method is provided in the Supplementary Material.

### Targeted Metabolites Analysis

Targeted metabolites from the phloem sap were determined by a high-throughput and multiplexed LC/MS/MRM method (Wei et al., 2010) with detail in the Supplementary Material. Metabolomics profiling was analyzed using a UPLC-ESI-Q-TRAP-MS system (UHPLC, 1290 Infinity LC, Agilent Technologies, Santa Clara, CA, United States) coupled with QTRAP 5500 (AB SCIEX, Framingham, MA, United States).

### Borate-Sucrose Complex Analysis in a Mixture Solution

The borate-sucrose complex in the mixture of boric acid (0.3 mM) and sucrose (300 mM) was determined by the ESI-Q-TOF-MS system in negative mode (ESI^–^). The detailed method of ESI-Q-TOF-MS was the same as the untargeted metabolite analysis.

### Borate-Sucrose Complex Analysis in Phloem Sap

Before testing the phloem sap, the mixture of boric acid and sucrose was analyzed by LC-MS to detect the peak time of the borate-sucrose complex. Then, the borate-sucrose complex in the phloem sap of T1- and T2-treated plants was determined by a UPLC-ESI-Q-TRAP-MS system in negative mode (ESI-). The detailed method of UPLC-ESI-Q-TRAP-MS was the same as the targeted metabolite analysis.

### Statistical Analysis

Unless specially stated, each value was expressed as the means ± standard deviation (SD) of four replications. Data analysis was performed by using independent-samples *t*-test (*P* < 0.05) or ANOVAs (Duncan test, *P* < 0.05) in SPSS for Windows 19.0 (SPSS Inc., Chicago, IL, United States).

## Results

### Comparison of ^10^B_pro_ and ^10^B_dff_ Among Leaves and Roots of T1, T2, and T3 Plants

The ^10^B_pro_ was at a similar level in either leaves (Figure 1B) or roots (Figure 1C) of T2 and T3 plants. But the ^10^B_pro_ in leaves (Figure 1B) or roots (Figure 1C) of both T2 and T3 plants were significantly higher than T1 plants. As for the ^10^B_dff_, it was significantly higher in the leaves of T3 plants than in the leaves of T2 plants (Figure 1D). However, no significant difference was observed for the ^10^B_dff_ between the roots of T2 and T3 plants (Figure 1E). Here, the ratio of root ^10^B_dff_ to leaf ^10^B_dff_ (RB_dff_/LB_dff_) was further calculated. Figure 1F showed that the RB_dff_/LB_dff_ ratio was significantly higher in T2 plants than T3 plants and the ratios in both T2 and T3 plants were significantly higher than T1 plants.

### Change of B_acc_ and ^10^B_dff_ in Leaves and Roots After Girdling or Shading

After girdling, the B_acc_ (Figure 2B) and the ^10^B_dff_ (Figure 2D) in leaves of T4 plants were significantly higher than those of T2 plants (control, without girdling). However, both B_acc_ (Figure 2C) and ^10^B_dff_ (Figure 2E) in roots of T4 plants were obviously lower than that of T2 plants. Moreover, ^10^B_dff_ was undetectable in the roots of T4 plants (Figure 2E).

On the other hand, shading significantly decreased the B_acc_ in roots of T5 plants compared to T2 plants (Figure 3C). A similar trend was also observed for the ^10^B_dff_ in the roots of T5 plants which was only 37% of T2 plants after shading (Figure 3E). Moreover, no significant difference in leaf B_acc_ (Figure 3B) or ^10^B_dff_ (Figure 3D) was observed between T2 and T5 plants.

### Change of Metabolites in the Phloem Sap of T2 Plants

**FIGURE 4:**
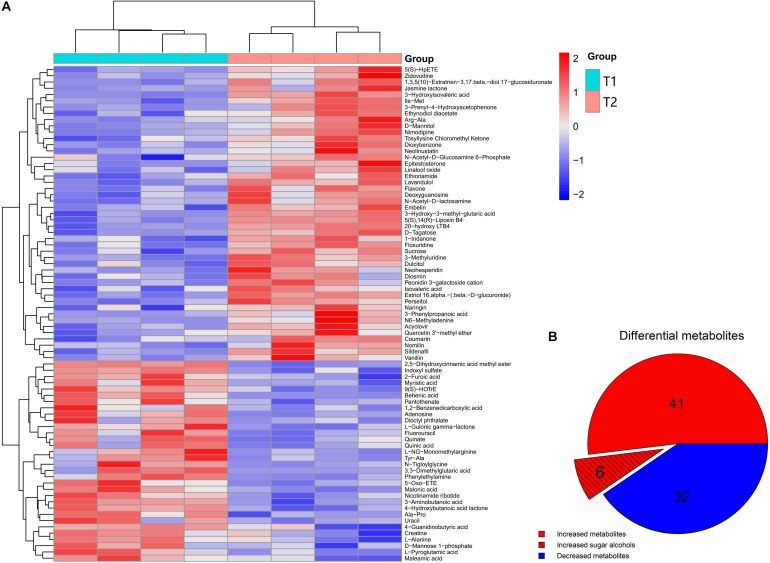
Unsupervised hierarchical clustering heat map of metabolites from the phloem sap of navel orange **(A)** and pie graph of the statistical analysis of differential metabolites **(B)**. Metabolites were compared as obtained between the phloem saps of foliar-water (T1) plants and foliar-^10^B (T2) plants.

**FIGURE 5:**
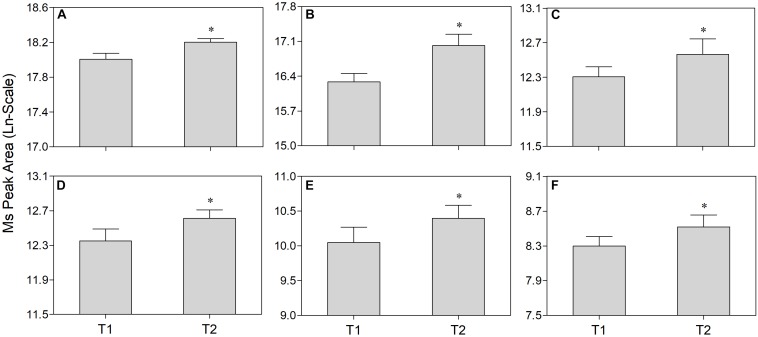
Comparison of sucrose **(A)**, tagatose **(B)**, mannitol **(C)**, dulicitol **(D)**, lavandulol **(E)**, and perseitol **(F)** concentration in the phloem sap of navel orange between foliar-water (T1) and foliar-^10^B (T2) treatments. “^∗^” indicates significant difference between T1 and T2 treatments (*t*-test, *n* = 4, *P* < 0.05).

Non-targeted metabolomics was performed to analyze differential metabolites in the phloem sap of T1 and T2 plants. PCA-QC (an unsupervised clustering method) analysis showed a significant difference in metabolites between the phloem sap of T1 and T2 plants with 49.5% and 52.0% total variance of the first two principal components in the positive and negative mode, respectively (Supplementary Figure S2). The heat map showed a significant change of metabolites that occurred in the phloem sap of T2 plants compared to T1 plants (Figure 4A). In detail, the phloem sap of T2 plants contained 47 and 32 metabolites significantly higher and lower, respectively, than those of T1 plants (Figure 4B). The increased metabolites in T2 plants mainly belonged to amino acids, esters, ketones, nucleosides, organic acids, sugars and sugar alcohols (Figure 4A). In them, six differential sugars and sugar alcohols (sucrose, tagatose, mannitol, dulicitol, lavandulol, and perseitol) were observed in the increased metabolites in the phloem sap of T2 plants (Figures 4A,B). To verify the authenticity of metabolomics analysis, they were selected to test Ms peak area between the phloem saps of T1 and T2 plants. It was found that their concentration in the phloem sap of T2 plants were significantly higher than those in T1 plants (Figure 5), which was consistent with the results of non-targeted metabolic analysis (Figure 4A). The concentration of sucrose was the highest among these sugars and sugar alcohols, and the Ln-peak area of sucrose was 18.05 and 18.20 in the phloem sap of T1 and T2 plants, respectively (Figure 5).

### Identification of Borate-Sucrose Complex in the Phloem Sap of T1 and T2 Plants

**FIGURE 6:**
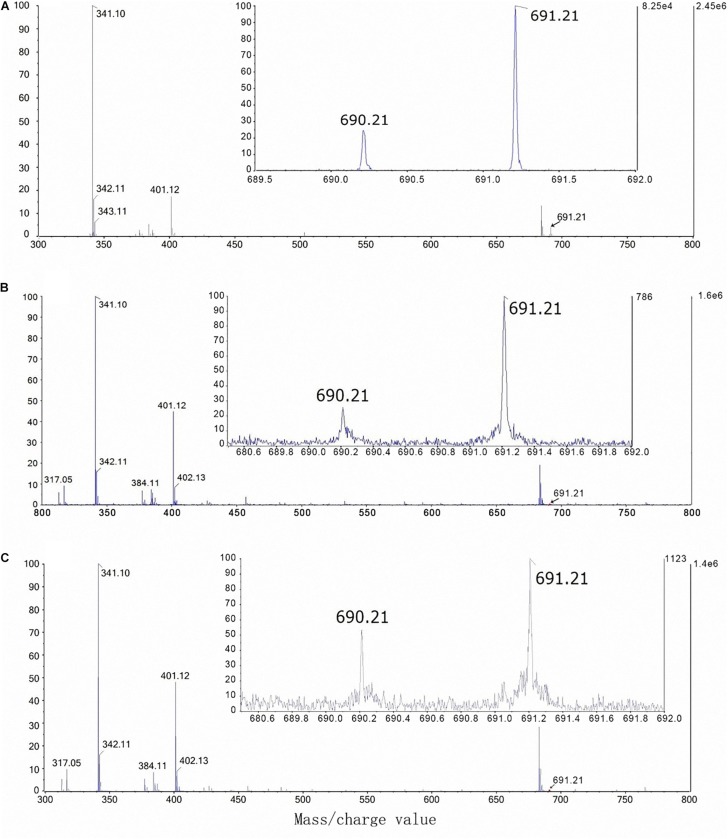
Borate-sucrose complex isolated from the artificial mixture of sucrose (300 mM) and boric acid (0.3 mM) by negative mode ESI-Q-TOF-MS **(A)**; from the phloem sap of foliar-water plants (T1) by negative mode UPLC-ESI-Q-TRAP-MS **(B)**; and from the phloem sap of foliar-^10^B plants (T2) by negative mode UPLC-ESI-Q-TRAP-MS **(C)**.

The mass/charge values of B-sucrose complexes [(C_24_H_40_O_22_)^10^B and (C_24_H_40_O_22_)^11^B] are 690.21 and 691.21, respectively (Table 1). The artificial mixture of sucrose (300 mM) and boric acid (0.3 mM) was first used to detect the borate-sucrose complex by using the ESI-Q-TOF-MS technique. Two ions with mass/charge values of 690.21 and 691.21 were observed in the negative mode profile of ESI-Q-TOF-MS analysis (Figure 6A). Moreover, the percentage value of (C_24_H_40_O_22_)^10^B intensity was nearly 25% of the (C_24_H_40_O_22_)^11^B intensity.

**TABLE 1:** Mass/charge values of sucrose-borate complexes determined by the ESI-Q-TOF-MS.

Formula	Mass/charge values	Description
(C_24_H_40_O_22_)^10^B	690.21	^10^Borate complex with 2 Suc
(C_24_H_40_O_22_)^11^B	691.21	^11^Borate complex with 2 Suc

On the other hand, after confirming the peak time of 690.21 and 691.21 mass/charge values (Supplementary Figure S3), the phloem sap of T1 (control) and T2 plants were also used to detect the mass/charge peak of B-sucrose complex by using the UPLC-ESI-Q-TRAP-MS technique. In the end, the mass/charge values of 690.21 and 691.21 were also observed in the phloem sap of T1 plants (Figure 6B) and T2 plants (Figure 6C). Moreover, the percentage value of (C_24_H_40_O_22_)^10^B intensity was nearly 25% of the (C_24_H_40_O_22_)^11^B intensity in the phloem sap of T1 plants (Figure 6B) while it was nearly 52% of the (C_24_H_40_O_22_)^11^B intensity in the phloem sap of T2 plants (Figure 6C).

## Discussion

### Foliar B Can Be Transported to the Roots Through the Phloem

Foliar application of fertilizers is an effective technique for sustainable production of crops and it may enhance the utilization of targeted tissues since nutrients can be simply supplied to the deficient tissues (Fernández and Eichert, 2009; Fernández and Brown, 2013). Boron is a vital micronutrient for healthy plant development (Wang et al., 2015), and foliar sprays of B have become a regular practice to rapidly remedy B deficiency in many plants, such as cranberry (*Vaccinium corymbosum*), grape (*Vitis vinifera*), and orange (*C. sinensis*) (DeMoranville and Deubert, 1987; Boaretto et al., 2011; Chormova et al., 2014). It can rapidly increase B concentrations in leaves and buds of subtropical plants (Eichert and Goldbach, 2010). Moreover, foliar B can be transported to some vegetative and reproductive organs (Huang et al., 2001; Stangoulis et al., 2001; Liu et al., 2012). In this study, by using ^10^B as tracer, we found that more ^10^B could be detected in the roots (Figure 1) and the ^10^B_pro_ (Figure 1C) or ^10^B_dff_ (Figure 1E) was significantly higher in foliar-^10^B treated plant roots than in foliar-water treated plant roots. These results suggested that foliar supplied B can be long distance transported from leaves to roots in citrus plants. In addition, the present results indicated that the RB_dff_/LB_dff_ in B-deficient plants (T2) was dramatically higher than in B-sufficient plants (T3) (Figure 1F), implying that more B could be transported to the roots under B-deficiency conditions. These findings support the hypothesis that the amount of B distribution depends on its status in plant tissues or organs (Shelp et al., 1996; Liakopoulos et al., 2009; Liu et al., 2012).

The transport of root absorbed B to different tissues or organs occurs through the xylem (Takano et al., 2008). However, few studies showed that foliar B can be transported and distributed to adjacent tissues or organs through the phloem (Brown and Shelp, 1997; Dannel et al., 2000; Pfeffer et al., 2001; Eichert and Goldbach, 2010). Girdling refers to removing a ring of bark or phloem. When carried out around the trunk, it has the immediate effect of blocking phloem-transported metabolites across the girdle (Wang et al., 2010; Appel et al., 2012). This technique is always used to detect whether the transport of a metabolite is or not through the phloem (De Schepper et al., 2013; Savage et al., 2016). In this study, girdling significantly increased B_acc_ (Figure 2A) and ^10^B_dff_ (Figure 2D) in leaves, while significantly decreased B_acc_ in roots (Figure 2C). Moreover, ^10^B_dff_ was undetectable in the roots of girdled plants (Figure 2D). These results strongly demonstrate that the transport of foliar B to roots is through the phloem.

### B-Sucrose Complex Plays a Key Role in B Basipetal Transport to the Roots

To date, the mechanisms for B transport in the xylem and its subsequent distribution have been associated with passive diffusion of boric acid, facilitated diffusion of boric acid via channels, and export of borate anion via transporters (Reid, 2014; Yoshinari and Takano, 2017). On the other hand, some reports suggest that foliar applied B can be transported to adjacent tissues or organs through the phloem by forming a complex with metabolites (Brown and Shelp, 1997; Reid, 2014). These metabolites were possibly related to some photosynthetic assimilates, such as polyols (Lehto et al., 2004; Will et al., 2011) and sucrose (Stangoulis et al., 2010). In citrus plants, sucrose is the main transportable photosynthetic assimilate (Dinant et al., 2010; Liu et al., 2015; Konrad et al., 2018). Moreover, shading has been proven to decrease carbohydrate production by limiting the photosynthesis of leaves (Osaki et al., 1995; Mäkelä et al., 2005; Correia et al., 2006). Here, we found that, when shading the plants with a black plastic net, the photosynthesis rate and photosynthetic active radiation (PAR) were significantly reduced (Supplementary Table S1). Moreover, the B_acc_ (Figure 3C) or ^10^B_dff_ (Figure 3E) of roots were significantly decreased (corresponding to 65% or 50% of control plants, respectively), providing evidence for the role of photosynthetic assimilates in the process of foliar B transport to roots through the phloem. On the other hand, the concentration of sucrose, tagatose, mannitol, dulcitol, perseitol, and lavandulol in the phloem sap was significantly increased after foliar ^10^B application compared to those of control plants (foliar water application) (Figures 4, 5). These results further indicate that scion photosynthetic assimilates are involved in the transport of foliar applied B from shoots to roots.

To confirm the possible role of sucrose in foliar B transport to the roots, we first conducted an *in vitro* experiment and obtained consistent results which are in line with the findings by Stangoulis et al. (2010). Namely, one borate molecule (^10^Borate or ^11^Borate) can form a complex with two sucrose molecules with the mass/charge value of 690.12 or 691.21 (Table 1 and Figure 6A). Subsequent *in vivo* analysis showed that B-sucrose complexes were also found in the phloem sap of control plants (T1) and foliar ^10^B-treated plants (T2) (Figures 6B,C), confirming that B transport in the phloem at least occurs by forming a complex with sucrose. Moreover, the percentage value of (C_24_H_40_O_22_)^10^B intensity was nearly 52% of the (C_24_H_40_O_22_)^11^B intensity in the phloem sap of foliar ^10^B-treated plants (Figure 6C), while it was nearly 25% in the phloem sap of control plants (Figure 6B), further showing that more ^10^B-sucrose complex existed in the phloem sap and thus more B can be translocated through the phloem and be distributed to the roots (Figure 1C).

Besides sucrose, some sugar alcohols also play a role in forming a complex with B (Hu et al., 1997; Hijaz and Killiny, 2014; Killiny et al., 2017). In this study, the concentration of tagatose, mannitol, dulcitol, perseitol, and lavandulol were also significantly higher in the phloem sap of foliar ^10^B-treated plants (T2) than in control plants (T1), although their concentration were lower than the concentration of sucrose (Figures 4, 5). However, we failed to identify their B complexes in the phloem sap, possibly due to the low concentration of such potential borate complexes, but their determination should be attempted in future trials.

## Conclusion

In this study, ^10^B foliar application experiment, together with girdling and shading treatments to citrus seedlings proved that foliar supplied B can be transported from leaves to roots via phloem in this species. The translocation of foliar supplied B to the roots is affected by plant B status and the synthesis of photosynthetic assimilates. Moreover, foliar supplied B can be transported and/or translocated into the roots through the phloem, at least by forming the B-sucrose complex. Overall, this study contributes to raise the knowledge on foliar-B fertilization and improves our understanding of the mechanisms of foliar B transport from shoots to roots in citrus.

## Data Availability Statement

The raw data supporting the conclusions of this article will be made available by the authors, without undue reservation, to any qualified researcher.

## Author Contributions

WD, Y-ZL, and S-AP designed the experiments. WD conceived the project, analyzed the data, wrote the article with contributions of all the authors, and prepared the experimental materials. Z-XH extracted the phloem sap from materials. Z-YP, SH, and S-AP provided technical and writing assistance. Y-ZL supervised and complemented the writing.

## Conflict of Interest

The authors declare that the research was conducted in the absence of any commercial or financial relationships that could be construed as a potential conflict of interest.
