# Supplementary material for: Foliar Supplied Boron Can Be Transported to Roots as a Boron-Sucrose Complex via Phloem in Citrus Trees
Source: Front Plant Sci. 2020 Mar 10;11:250. doi: 10.3389/fpls.2020.00250 (PMC7076173; doi:10.3389/fpls.2020.00250)
Supplement: Supplementary file 1 [file Presentation_1.pdf]

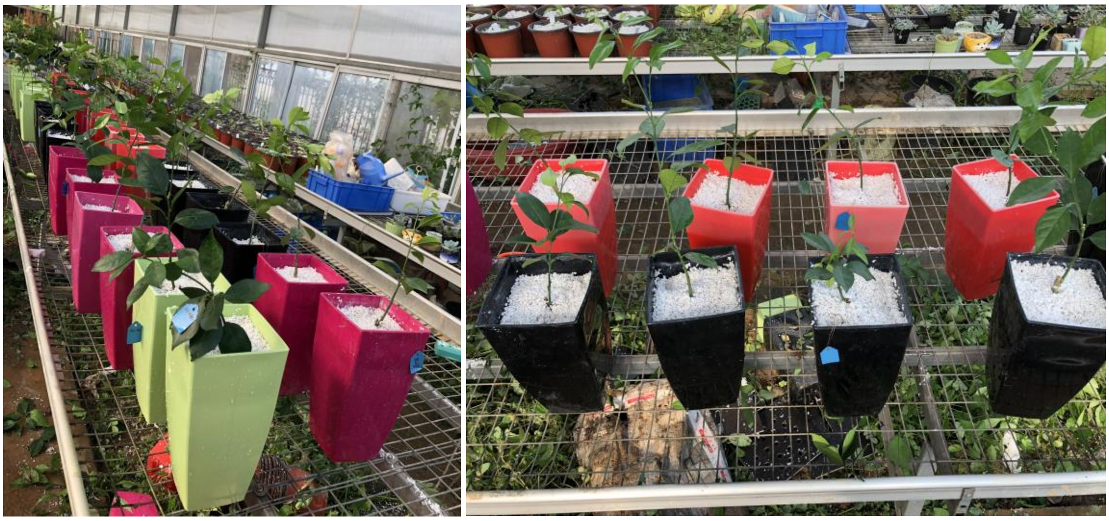

Fig. S1 Pictures of ‘Newhall’ grafted plants that were used for experiments.

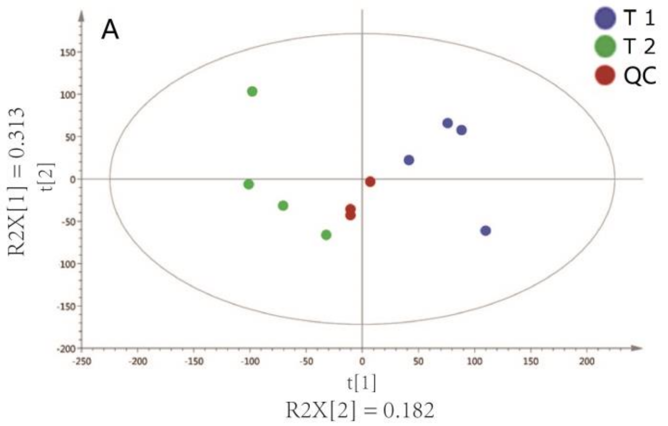

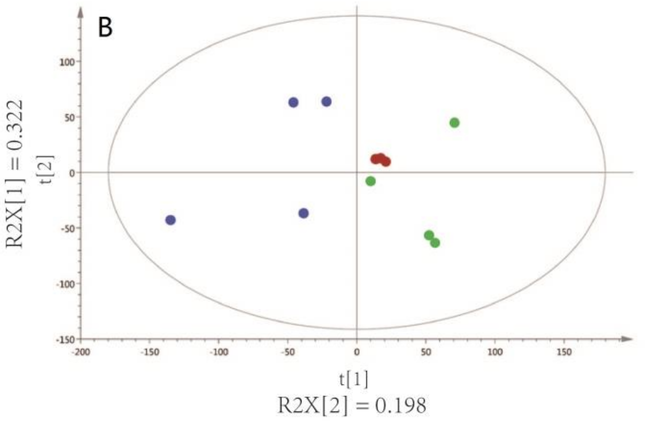

Fig. S2 PCA-QC score plots of phloem sap samples, derived from UPLC-ESI-Q-TOF-MS spectra concerning the foliar water (T1, blue dot) and foliar-B (T2, green dot) treatments in the positive mode (A) and negative mode (B).

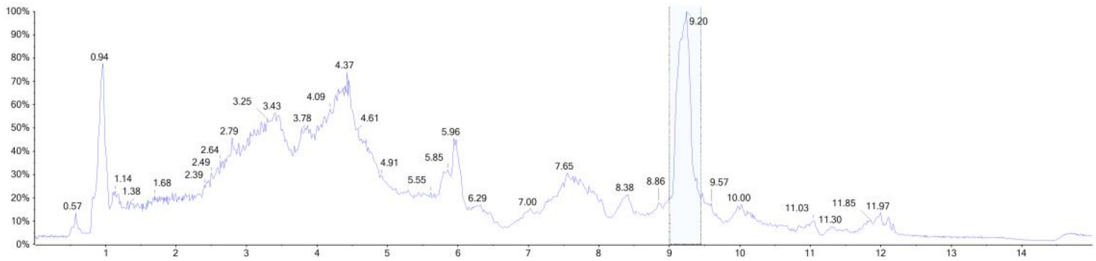

Fig. S3 Total ion chromatogram (TIC) of sucrose-borate complex determined by UPLC-ESI-Q-TRAP-MS.

Table S1 Comparison of photosynthesis, leaf temperature, external PAR and sample cell CO_2 between T2 and T5 treatments. Values are means of four replicates±SD. "*" denotes significant differences between T2 and T5 treatments according to the t test ($P < 0.05$).

Treatments	Photosynthesis (μ mol·$m^{-2}s^{-1}$)	Leaf temperature (℃)	External PAR (μ mol·$m^{-2}s^{-1}$)	Sample cell CO_2 (μ mol·mol^{-1})
T2	4.93±0.10*	30.52±0.36	221.34±20.09*	446.45±16.01
T5	3.12±0.07	30.91±0.28	15.20±2.14	440.68±12.12

UPLC-ESI-Q-TOF-MS analyses

For hydrophilic interaction liquid chromatography (HILIC) separation, samples were analyzed using a 2.1 mm × 100 mm ACQUIY UPLC BEH 1.7 µm column (Waters, Ireland). The flow rate was 0.5 mL/min and the mobile phase contained: A = 25 mM ammonium acetate and 25 mM ammonium hydroxide in water and B = acetonitrile (ACN). The gradient was 95% B for 0.5 min and was linearly reduced to 65% in 6.5 min, and then reduced to 40% in 2 min and maintained for 1 min, and then increased to 95% in 1.1 min, with 5 min re-equilibration period employed. Both electrospray ionization (ESI) positive-mode and negative mode were applied for MS data acquisition. The ESI source conditions were set as follows: Ion Source Gas 1 as 60, Ion Source Gas 2 as 60, curtain gas as 30, source temperature: 600 °C, IonSpray Voltage Floating (ISVF) ± 5500 V. In MS only acquisition, the instrument was set to acquire over the m/z range 60-1200 Da, and the accumulation time for TOF MS scanning was set at 0.15 s/spectra. In auto MS/MS acquisition, the instrument was set to acquire over the m/z range 25-1200 Da, and the accumulation time for product- ion scan was set at 0.03 s/spectra. The product-ion scan was acquired using information-dependent acquisition with a high sensitivity mode selected. The collisional energy was fixed at 30 V with ± 15 eV. Declustering potential was set as ± 60 V. Quality control (QC) samples were prepared by pooling aliquots of all samples that were representative of the samples under analysis, and used for data normalization. Blank samples (75 %ACN in water) and QC samples were injected during acquisition.

Multivariate statistical analysis

SIMCAP software (Version 14.0, Umetrics, Umeå, Sweden) was used for all multivariate data analyses and modeling. Data were mean-centered using Pareto scaling. Models were built on the principal component analysis (PCA), orthogonal partial least-square discriminant analysis (PLS-DA) and partial least-square discriminant analysis (OPLS-DA). All the models evaluated were tested for overfitting with methods of permutation tests. The descriptive performance of the models was determined by R2X (cumulative) (perfect model: R2X (cum) = 1) and

R2Y (cumulative) (perfect model: R2Y (cum) = 1) values while their prediction performance was measured by Q2 (cumulative) (perfect model: Q2 (cum) = 1) and a permutation test (n = 200). The permuted model should not be able to predict classes: R2 and Q2 values at the Y-axis intercept must be lower than those of Q2 and the R2 of the non-permuted model. OPLS-DA allowed the determination of discriminating metabolites using the variable importance on projection (VIP). The VIP score value indicates the contribution of a variable to the discrimination between all the classes of samples. Mathematically, these scores are calculated for each variable as a weighted sum of squares of PLS weights. The mean VIP value is 1, and usually VIP values over 1 are considered as significant. A high score is in agreement with a strong discriminatory ability and thus constitutes a criterion for the selection of biomarkers.

The discriminating metabolites were obtained using a statistically significant threshold of variable influence on projection (VIP) values obtained from the OPLS-DA model and two-tailed Student's t-test (p-value) on the normalized raw data at univariate analysis level. The p-value was calculated by one-way analysis of variance (ANOVA) for multiple groups analysis. Metabolites with VIP values greater than 1.0 and a p-value less than 0.05 were considered to be statistically significant metabolites. Fold change was calculated as the logarithm of the average mass response (area) ratio between two arbitrary classes. On the other side, the identified differential metabolites were used to perform cluster analyses with R package.

UPLC-ESI-Q-TRAP-MS analyses

The LC-MS/MS portion of the platform was based on a UHPLC system (1290 series, Agilent Technologies) equipped with an ACQUITY UPLC BEH Amide column (1.7 μm, 2.1 mm×100 mm, Wasters) and a triple quadruple mass spectrometer (5500 QTRAP, AB SCIEX) in the multiple reaction monitoring (MRM) mode. Metabolites were detected in electrospray negative-ionization and positive-ionization mode. The 2 μL samples were injected sequentially. The ACQUITY UPLC BEH Amide column (1.7 μm, 2.1 mm×100 mm, Wasters) was heated to 45 °C under a flow rate of 300 μL/min. A gradient was used to separate the compounds consisted of solvent A (20

mM ammonium acetate, 50% acetonitrile, pH 9.5) and solvent B (100% acetonitrile). The gradient started at 5% solvent A for 1 min and increasing linearly to 35% solvent A over 11 min, and then increased linearly to 60% solvent A over 2 min, and then decreasing to 5% of solvent A in 2 min, then keeping for extra 2 min. QC samples were injected during acquisition.

The MS conditions were set as follows: Source Temperature 550℃, Ion Source Gas1(GAS1): 40, Ion Source Gas2(GAS2): 50, Curtain Gas(CUR): 35, Ion Spray Voltage Floating (ISVF) ：-4500V；The mass spectrometer was operated with a dwell time of 200 ms. To construct the metabolite MRM library, each metabolite standard (50 mg/mL) was first analyzed by LC-MS/MS to get the optimal MRM transition parameters. Then the retention time of each metabolite was determined by measuring the corresponding MRM (Q1/Q3) transition individually. A standard mixture sample (6 standard mixtures) that contains all of the 6 metabolites was measured together and used as the reference peaks for metabolite identification. Raw MRM data files were processed by peak finding, alignment, and filtering using MultiQuant software.
